# Thinking flexibly: A cognitive remediation therapy–informed intervention for autistic youth

**DOI:** 10.1177/13623613251407294

**Published:** 2025-12-31

**Authors:** Jasmin Dipre, Lauren Burton, Kate Tchanturia, Matthew J Hollocks

**Affiliations:** 1 King’s College London, UK; 2 South London and Maudsley NHS Foundation Trust, UK

**Keywords:** anxiety, autism spectrum disorder, depression, executive functions, treatment

## Abstract

Autistic people are known to demonstrate greater difficulties with cognitive flexibility. These difficulties are associated with a range of poor outcomes, including elevated rates of anxiety and depression. Current intervention approaches fail to account for these specific mechanisms. In this study, we address this by piloting a new intervention which aims to specifically target cognitive inflexibility. The ‘Thinking Flexibly’ intervention has been co-designed with autistic youth based on previous work, including cognitive remediation therapy. Twenty autistic adolescents were recruited from clinical services and offered the eight-session intervention. Acceptability was assessed through participant drop-out and session completion. Participants (and their parents) also completed questionnaire measures of cognitive inflexibility, anxiety, depression and functional outcomes before and after the intervention. Our results indicated good acceptability with 19/20 participants completing the intervention and session attendance was high, with a single missed session. We found significant increases in flexibility across both parent- and self-report of a moderate effect. Participants reported significant reductions in symptoms of anxiety and depression. We conclude that ‘Thinking Flexibly’ is acceptable for use with autistic adolescents and shows promise as a standalone or adjunct therapy. Changes on outcome measures are promising and warrant further investigation in a larger trial.

**Lay abstract:**

Autistic people often have greater difficulties with flexible thinking when compared to those without autism. People with difficulties in this area often appear to be ‘stuck’ in their style of thinking or in their way of behaving. This can have a negative impact on several areas of life, including on their mental health. In this study, we developed and tested an intervention designed to help autistic individual think flexibly. We showed that the intervention was acceptable (people were able to attend and complete the intervention) and some improvements in cognitive flexibility and mental health symptoms were reported.

## Introduction

Autistic young people are at disproportionate risk for having co-occurring mental health difficulties. Anxiety disorders are particularly prominent with 52.5% of adolescents having at least one *Diagnostic and Statistical Manual of Mental Disorders* (5th ed.; DSM-5) anxiety diagnosis (Hollocks, Leno, et al., 2023). Depression is also present in around 10% of cases, almost double the rate of the general youth population. In addition, autistic youth are known to demonstrate greater cognitive inflexibility (CI), defined as a reduction in the ability to easily switch between thoughts and behaviours, resulting in the inability to engage in more adaptive behavioural strategies. Worse CI has been implicated as a key mechanism associated with both increased anxiety and depression in autistic youth (Hollocks et al., 2022; Lei et al., 2022; Ozsivadjian et al., 2021). In addition to worse mental health, CI has also been associated with social difficulties (Berger et al., 2003) and poor adaptive functioning (Wallace et al., 2016). This suggests that interventions which can improve CI may have a wide-ranging impact on functioning, including important effects for reducing mental health difficulties for autistic people.

Existing approaches to psychological intervention for mental health difficulties, such as anxiety and depression experienced by autistic people (i.e. cognitive behavioral therapy (CBT)), make adaptations to increase treatment accessibility (Cooper et al., 2018). While these have shown some positive outcomes, high levels of CI is a notable indicator of poor response to psychological interventions, particularly CBT, due to the need to flexibly alter patterns of thoughts or behaviours. No current interventions are designed to target specific cognitive features associated with a diagnosis of autism, such as CI, to improve outcome.

Cognitive remediation therapy (CRT) is an established intervention targeting cognitive processes strongly associated with a diagnosis of autism, including CI. CRT is an intervention, which encourages patients to become aware of their thinking process and provide alternative strategies. CRT also encourages the use of within session experimentation with different ways of thinking and behaving, which can then be reflected upon during the next session (Tchanturia et al., 2017). CRT has shown promise in improving these cognitive difficulties in adults with anorexia nervosa and autistic features (Dandil et al., 2020) and has been shown to have a very low drop-out rate (10%–15%; Tchanturia et al., 2014). Together this strongly suggests that if fully adapted for autistic youth it could be an effective and suitable intervention for those with complex mental health difficulties.

The objective of the current study was to co-design a version of CRT suitable for use with autistic adolescents and then evaluate the acceptability of the intervention (drop-out rates; session attendance) and to gather preliminary data on clinical outcome measures to determine the intervention’s potential to increase flexible thinking and reduce symptoms of anxiety and depression.

## Methods

### Participants

Twenty autistic youth (mean age = 14 years 3 months; range = 12 years 2 months–16 years 5 months) were recruited from child and adolescent mental health services in South London. Most participants were recruited from intervention waiting lists and this is reflected in the level of difficulties experienced by our sample who have several co-occurring conditions (anxiety disorder 7; attention-deficit hyperactivity disorder (ADHD) = 6 (with 5 more awaiting assessment for ADHD)). See Table 1 for participant characteristics. The study protocol underwent ethical review by the London-Brent Research Ethics Committee – 24/LO/0224.

**Table 1.** Descriptive statistics.

Descriptive variables			
Age (mean (range))	14 years, 3 months (12 years, 2 months – 16 years 5 months)		
Gender, *N* (%)			
Female	7 (35%)		
Male	12 (60%)		
Transmale	1 (5%)		
Ethnicity, *N* (%)			
White	15 (75%)		
Black	3 (15%)		
Mixed race	2 (10%)		
Co-occurring conditions, *N* (%)			
Anxiety disorders	7 (35%)		
Depressive disorders	5 (25%)		
ADHD	6 (30%)		
Of which are medicated	2 (10%)		
Questionnaire measures (mean; range)		Baseline	Post-treatment
Flexibility Scale – Self		40.8 (22–64)	34.7 (19–50)
Flexibility Scale – Parent		51.5 (29–76)	46.9 (17–72)
DFlex		52.3 (40–72)	49.5 (32–61)
RCADS Anxiety – Self		61.4 (33–80)	53.2 (34–80)
RCADS Anxiety – Parent		69.6 (41–80)	68.2 (46–80)
MFQ – Self		29.7 (2–58)	22.2 (8–41)
MFQ – Parent		22.3 (4–42)	17.9 (5–33)
WSAS – Self		19.6 (5–34)	14.7 (2–30)
WSAS – Parent		23.5 (11–33)	21.6 (8–32)

FS-SR: Flexibility Scale – Self-report; DFlex: Detail and Flexibility Scale – Flexibility subscale; RCADS: Revised Child Anxiety and Depression Scale; MFC: Mood and Feelings Questionnaire; WSAS: Work and Social Adjustment Scale.

### Design

This was a single-arm intervention design. All participants were invited to take part in the eight-session ‘Thinking Flexibly’ adapted CRT intervention (see below). All clinical outcome measures were collected pre- and post-intervention and are detailed below.

### The ‘Thinking Flexibly’ intervention

The intervention structure and material were adapted following co-design with autistic young people from a group-based CRT intervention for adolescence (https://www.katetchanturia.com/_files/ugd/2e1018_bb804c6eeca3421d98e4fb29f20dea1e.pdf). Sessions were held on average twice weekly over a 4- to 6-week period. Sessions included one therapist and one autistic young person, and we flexibly allowed parents to join sessions if required or requested (by the young person). The session content covered five main themes: (1) psychoeducation around CI and its impact/goal setting (one session); (2) bigger picture thinking (two sessions); (3) thinking flexibly (two sessions); (4) multitasking (one session) and (5) perfectionism (one session), followed by a final session focused on consolidating learning from sessions 1 to 7. Each session followed a similar structure, starting with the review of the previous session/between session work, then one or two structured activities designed to generate discussion and refection on the impact of CI on day-to-day life. These reflections are then used by the therapist to make links to the young person’s individual goals, areas of strength and difficulty experienced by the young person and to set behaviour change goals to be completed between sessions.

### Measures of acceptability and feasibility

Acceptability of this intervention was assessed using participant retention and drop-out rates, between session homework completion. Completion rates for both parent and self-report clinical outcome measures were used as a secondary measure of acceptability and were completed before and after the intervention was complete.

### Clinical outcomes

#### Flexibility Scale, youth and parent versions

The Flexibility Scale (FS) consists of 27 items which measure different elements of CI. Each item is scored on a 4-point Likert-type scale (from 0 = No to 3 = Always), with a higher score on each subscale representing lower flexibility. The FS has recently been adapted for use as a self-report scale and consists of 24 items (Hollocks, McQuaid, et al., 2023).

#### Detail and Flexibility Questionnaire – cognitive rigidity subscale

The Detail and Flexibility Questionnaire (DFlex; Roberts et al., 2011) is a 24-item self-report scale measuring two aspects of neurocognitive functioning: cognitive rigidity (difficulty with set-shifting/flexibility) and attention to detail (weak coherence). For this study, we have only included the 12-item cognitive rigidity subscale *as a secondary brief self-report measure of CI.*

#### Revised Child Anxiety and Depression Scale, youth and parent versions

The Revised Child Anxiety and Depression Scale (RCADS; Chorpita et al., 2005) is a measure for children aged 8–18, providing both an anxiety total score and ﬁve subscale *T*-scores (separation, generalised anxiety disorder, panic, social phobia, obsessions/compulsions and depression). In this study, parent and youth-reported anxiety symptoms were measured using the anxiety total scores. *T*-scores adjusted for school grade have been calculated, where a *T*-score of > 70 is indicative of symptoms in the clinical range.

#### Mood and Feelings Questionnaire, youth and parent versions

The Mood and Feelings Questionnaire (MFQ; Angold et al., 1995) consists of a series of 33 descriptive phrases regarding how the subject has been feeling or acting recently and is a screening tool for depression in children and young people aged 6–19. The MFQ is included in this study as secondary brief measure of mood and depression symptoms.

#### Work and Social Adjustment Scale, Youth and Parent versions

The Work and Social Adjustment Scale (WSAS)–Y/P (Jassi et al., 2020) is a brief global measure of functional impairment and measures difficulties in school and employment (e.g. summer jobs), everyday activities (e.g. personal hygiene, helping out at home), social activities (e.g. going out with friends), leisure time (e.g. reading, playing videogames) and family/relationships (parents, siblings, girlfriends/boyfriends). For each item, the individual is asked how much their problem (or their child’s problem) impairs their (or their child’s) ability to carry out the activity, with responses ranging from ‘Not at all’ (0) to ‘Severely impaired’ (8), generate a total score ranging from 0 to 40, with higher scores indicating higher impairment.

#### Statistical analyses

Measures of acceptability such as drop-out rate, session attendance and therapy adherence, and responses are described descriptively. Outcomes across clinical measures are presented descriptively and group mean scores pre- and post-intervention will be compared using paired-samples *t*-tests and effect size estimated using Hedges’ *g*. As this is a pilot study, a formal sample size calculation is not recommended. We followed guidelines that recommend a minimum sample size of 12 to 30 per group (Julious, 2005; Sim & Lewis, 2012).

## Results

### Acceptability

Of the 20 participants originally enrolled in the group, 19 participants completed the intervention (95% completion rate). The one young person who decided to no longer take part in the intervention terminated prematurely prior to the third session. This individual who withdrew did so due to family bereavement. Session attendance was high, with a single missed session and 18/19 participants completing all sessions. If clinicians facilitating the session were informed in advance of non-attendance with reasons including physical illness, pre-arranged family commitments, receiving an after-school detention and difficulties with transport when attempting to arrange travel to the clinic, an alternative session time was offered, and this was not recorded as a missed session. Outcome completion rate for the 19 participants who completed the intervention was 100%. Completion of formal between session work was 42%. It was noted that completion of activities was higher but young people did not complete written worksheets.

### Clinical outcomes

**Figure 1.**
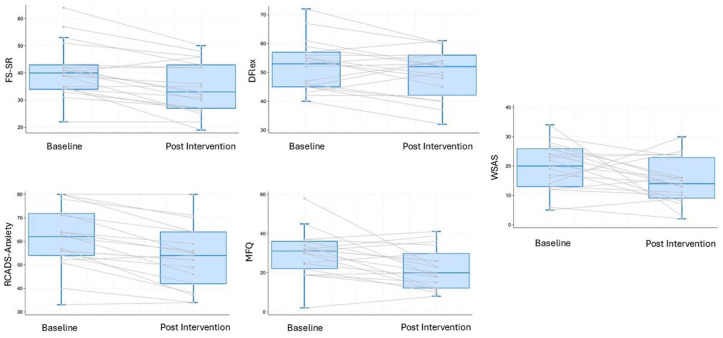
Group and Individual Change on Key Self-Reported Clinical Outcome Measures. FS-SR: Flexibility Scale – Self-report; DFlex: Detail and Flexibility Scale – Flexibility subscale; RCADS: Revised Child Anxiety and Depression Scale; MFQ: Mood and Feelings Questionnaire; WSAS: Work and Social Adjustment Scale.

Paired-samples *t*-tests were conducted to contrast pre- and post-intervention scores across all outcomes (see Figure 1). Both the self- and parent-reported FS scores showed significant reductions post intervention with a medium effect size (FS-self-report: *t*(18) = 2.38, *p* = 0.028, *g* = 0.62; FS parent report: *t*(18) = 4.60, *p* < 0.001, *g* = 0.59). Differences in our secondary measure of CI, the DFlex cognitive rigidity subscale, did not reach significance (*t* = 1.89, *p* = 0.074, *g* = 0.24). The RCADS-total anxiety score showed differences in self-reported anxiety symptoms with a medium effect size (*t*(18) = 5.62, *p* = 0.012, *g* = 0.67), but not on parent-report (*t* (18) = 0.63, *p* = 0.53, *g* = 0.12). Similarly, both the MFQ (Self-report: *t*(18) = 2.78, *p* < 0.001, *g* = 0.69; parent-report: *t*(18) = 1.90, *p* = 0.07, *g* = 0.52) and the WSAS (Self-report: *t*(18) = 2.08, *p* = 0.05, *g* = 0.63; parent-report: *t*(18) = 1.5, *p* = 0.13, *g* = 0.31) showed differences in self-reported but not parent-reported outcomes.

## Discussion

In this study, we have found the acceptability of the ‘Thinking Flexibly’ intervention for autistic youth. We have also presented some preliminary evidence to suggest that this intervention acts to increase flexible thinking and reduces self-reported symptoms of anxiety and depression.

The primary purpose of this study was ensuring the intervention, having been co-designed with a small group of autistic young people, was felt to be acceptable when used in a clinical sample. Having only a single drop-out due to personal circumstances is strongly indicative of high acceptability. If replicated, this effective completion rate of 95% is higher than reported in trials of adapted CBT for anxiety which tend to be at or around 85%–90% (Wood et al., 2015). The latter has a much higher number of sessions, but even in a low-intensity five-session intervention of depression in autistic adults, the authors report completion rates of 85%, with 71% completing all sessions (Russell et al., 2020). In contrast, we found that 18/19 completed all sessions, with only one session being missed across all participants.

In addition to good acceptability, we also found shifts across key outcome measures, including reductions of a moderate effect in both self- and parent-reported CI. This is indicative of significant improvements in our primary outcome that warrants further evaluation. Importantly, there were also reductions in self-reported anxiety and depression, with moderate-to-high and moderate effect sizes, respectively. This supports the premise that CI is a key process associated with anxiety and depression for autistic people (Lei et al., 2022), and intervening in this way may be beneficial. It is important to note that while anxiety and depression are not explicitly addressed in the intervention, many examples of flexible/inflexible behaviour (e.g. sticking to a specific routine) described by the young people were associated with anxiety. Therefore, by working on these behaviours during the intervention symptoms of anxiety and low mood are indirectly being targeted. We hypothesise that our high levels of engagement, indicated by low drop out and high session attendance, may have been supported by not focusing explicitly on emotions. Clinically, we frequently observe disengagement from therapy when emotions are initially discussed, possibly related to difficulties around alexithymia of challenges with managing difficulty emotions (Mazefsky et al., 2013). Our findings of positive changes in functional outcomes are also positive suggesting that the intervention may lead to better engagement with everyday activities.

It should be noted that the parent-reported anxiety and depression scales did not indicate any significant symptom reduction. As with the positive findings, this needs to be interpreted within the context of a small sample, but it could also be expected that observable changes related to internalising symptoms may take longer to emerge. This suggests that any future trial testing the intervention includes a period of follow-up. This is one of the key limitations of the current pilot, which we plan to address in a future study. Future evaluation of the thinking flexibly intervention could also benefit from an objective neuropsychological measure of CI to compliment behavioural report.

In conclusion, ‘Thinking Flexibly’ is a promising and acceptable intervention, which following further evaluation may provide an alternative, or an adjunct approach to the treatment of common difficulties in daily living and mental health experienced by autistic people. Our positive findings indicate that further evaluation of the intervention, in a larger study with a randomised controlled trial design, is warranted.
